# Surgical left atrial appendage occlusion in valvular heart disease without atrial fibrillation: the OPINION trial

**DOI:** 10.1093/eurheartj/ehaf674

**Published:** 2025-09-01

**Authors:** Xin Yuan, Fan Ju, Hengchao Wu, Yanyan Zhao, Xin Wang, Sheng Liu, Xianqiang Wang, Junming Zhu, Pixiong Su, Fei Xu, Wei Feng, Yan Yang, Yang Wang, Hansong Sun

**Affiliations:** Department of Cardiovascular Surgery, Fuwai Hospital, Chinese Academy of Medical Sciences and Peking Union Medical College, National Centre for Cardiovascular Diseases, No. 167 North Lishi Road, Xicheng District, Beijing 100037, China; Department of Cardiovascular Surgery, Fuwai Hospital, Chinese Academy of Medical Sciences and Peking Union Medical College, National Centre for Cardiovascular Diseases, No. 167 North Lishi Road, Xicheng District, Beijing 100037, China; Department of Cardiovascular Surgery, Fuwai Hospital, Chinese Academy of Medical Sciences and Peking Union Medical College, National Centre for Cardiovascular Diseases, No. 167 North Lishi Road, Xicheng District, Beijing 100037, China; Medical Research and Biometrics Centre, Fuwai Hospital, Chinese Academy of Medical Sciences and Peking Union Medical College, National Centre for Cardiovascular Diseases, No. 167 North Lishi Road, Xicheng District, Beijing 100037, China; Department of Cardiovascular Surgery, Fuwai Hospital, Chinese Academy of Medical Sciences and Peking Union Medical College, National Centre for Cardiovascular Diseases, No. 167 North Lishi Road, Xicheng District, Beijing 100037, China; Department of Cardiovascular Surgery, Fuwai Hospital, Chinese Academy of Medical Sciences and Peking Union Medical College, National Centre for Cardiovascular Diseases, No. 167 North Lishi Road, Xicheng District, Beijing 100037, China; Department of Cardiovascular Surgery, Fuwai Hospital, Chinese Academy of Medical Sciences and Peking Union Medical College, National Centre for Cardiovascular Diseases, No. 167 North Lishi Road, Xicheng District, Beijing 100037, China; Department of Cardiovascular Surgery, Beijing Anzhen Hospital, Capital Medical University, No. 2 Anzhen Road, Chaoyang District, Beijing 100029, China; Department of Cardiac Surgery, Beijing Chaoyang Hospital, Capital Medical University, No. 8 Gongti South Road, Chaoyang District, Beijing 100020, China; Department of Cardiovascular Surgery, Fuwai Hospital, Chinese Academy of Medical Sciences and Peking Union Medical College, National Centre for Cardiovascular Diseases, No. 167 North Lishi Road, Xicheng District, Beijing 100037, China; Department of Cardiovascular Surgery, Fuwai Hospital, Chinese Academy of Medical Sciences and Peking Union Medical College, National Centre for Cardiovascular Diseases, No. 167 North Lishi Road, Xicheng District, Beijing 100037, China; Department of Cardiovascular Surgery, Fuwai Hospital, Chinese Academy of Medical Sciences and Peking Union Medical College, National Centre for Cardiovascular Diseases, No. 167 North Lishi Road, Xicheng District, Beijing 100037, China; Medical Research and Biometrics Centre, Fuwai Hospital, Chinese Academy of Medical Sciences and Peking Union Medical College, National Centre for Cardiovascular Diseases, No. 167 North Lishi Road, Xicheng District, Beijing 100037, China; Department of Cardiovascular Surgery, Fuwai Hospital, Chinese Academy of Medical Sciences and Peking Union Medical College, National Centre for Cardiovascular Diseases, No. 167 North Lishi Road, Xicheng District, Beijing 100037, China

**Keywords:** Valvular heart disease, Atrial fibrillation, CHA₂DS₂-VASc score, Surgical left atrial appendage occlusion, Thromboembolism

## Abstract

**Background and Aims:**

While surgical left atrial appendage occlusion (SLAAO) reduces stroke in atrial fibrillation (AF) patients, its efficacy in patients without pre-operative AF but with CHA₂DS₂-VASc ≥2 remains uncertain despite their high post-operative AF risk (15–54%). The aim of this study was to evaluate whether prophylactic SLAAO reduces post-operative thrombo-embolic events in valvular surgery patients.

**Methods:**

The OPINION was a multicentre, open-label, randomized, superiority trial conducted at three cardiac surgery centres in China. Eligible non-AF patients with CHA₂DS₂-VASc ≥2 and an indication for valve repair or replacement due to mitral or aortic valve lesions were randomly assigned (1:1) to undergo SLAAO (intervention arm) or not undergo SLAAO (control arm) during surgery. The primary outcome was a composite of ischaemic stroke, transient ischaemic attack (TIA), or cardiovascular mortality assessed at 1 year. The primary analysis was done in the intention-to-treat population.

**Results:**

Between April 2021 and June 2024, a total of 2157 patients were enrolled and randomized. After exclusion of 39 patients who withdrew informed consent, 2118 participants were included in the intention-to-treat population (1062 in the SLAAO group and 1056 in the control group). Baseline characteristics were well-balanced between the SLAAO group and control group (mean age 55.5 [11.4] vs 55.6 [11.5] years, *P* = .65; female 32.9% vs 32.3%, *P* = .78; CHA_2_DS_2_-VASc score 2.88 [0.98] vs 2.87 [0.96], *P* = .83; median EuroSCORE II 1.58% [1.42%] vs 1.56% [1.28%], *P* = .74). The 1-year primary endpoint occurred in 73 (6.9%) patients in the SLAAO group and in 87 (8.2%) patients in the control group (hazard ratio 0.83; 95% confidence interval 0.61–1.14; *P* = .25).

**Conclusions:**

For valvular surgery patients with CHA₂DS₂-VASc scores ≥2 but no pre-operative AF, routine prophylactic left atrial appendage closure did not significantly reduce the incidence of the primary composite endpoint (ischaemic stroke, TIA, and cardiovascular mortality) at 1-year follow-up.

**Trial Registration:**

ChiCTR.org registry ChiCTR2100042238

## Structured Graphical Abstract

**Structured graphical abstract:**
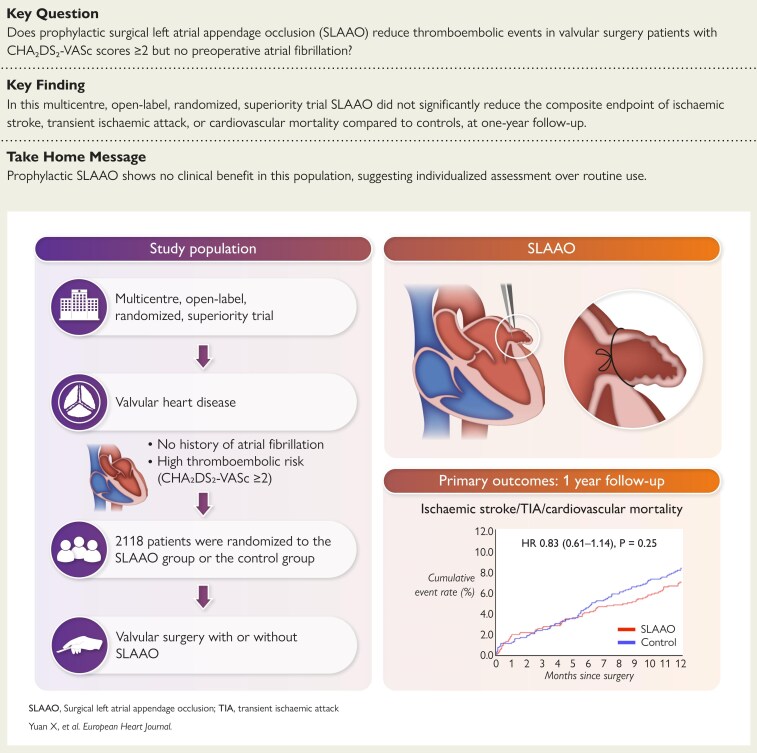
HR, hazard ratio; SLAAO, surgical left atrial appendage occlusion; TIA, transient ischaemic attack.

**See the editorial comment for this article ‘Routine left atrial appendage closure in non-AF patients: an unresolved issue’, by P. Kruger *et al*., https://doi.org/10.1093/eurheartj/ehaf882.**

## Introduction

Atrial fibrillation (AF) is a well-established risk factor for thrombo-embolic events and mortality following cardiac surgery,^1–8^ with the left atrial appendage (LAA) serving as the predominant source of thrombus formation.^9,10^ Substantial evidence from multiple studies has demonstrated that surgical LAA occlusion (SLAAO) significantly reduces long-term stroke risks in AF patients.^11^

Based on this evidence, the 2020 American College of Cardiology (ACC)/American Heart Association (AHA) valvular heart disease guidelines recommended that for patients with AF or atrial flutter who are undergoing valve surgery, LAA ligation/excision is reasonable to reduce the risk of thrombo-embolic events (Class 2a recommendation).^12^

However, the clinical benefits of prophylactic SLAAO remain uncertain for patients without pre-operative AF but with elevated thrombo-embolic risk (CHA₂DS₂-VASc score ≥2). This population demonstrates a significantly increased incidence of post-operative AF (POAF) which is related to both short- and long-term prognosis, with reported rates ranging from 15% to 54%.^13–16^ While theoretically SLAAO might reduce long-term thrombo-embolic events in these patients, existing studies have produced conflicting results—some suggesting potential benefits,^17–19^ whereas others have shown no significant differences.^20,21^ Importantly, high-quality randomized controlled trial evidence remains lacking. In summary, current evidence regarding the efficacy of SLAAO in this population remains inconclusive, with no consensus on its net clinical benefit when weighing thrombo-embolic prevention against procedural risks.

To address these evidence gaps, we conducted a multicentre randomized controlled trial to evaluate whether prophylactic SLAAO reduces post-operative thrombo-embolic events in valvular surgery patients without AF but with CHA₂DS₂-VASc scores ≥2, while simultaneously exploring potential beneficiary subgroups.

## Methods

### Trial design and oversight

The OPINION study was a multicentre, open-label, randomized, superiority trial. Three cardiovascular surgery centres in Beijing, China, participated in the study, including Fuwai Hospital, Beijing Anzhen Hospital, and Beijing Chaoyang Hospital. The ethics committee of Fuwai Hospital and the ethics committee of each participating site approved the trial. Prof. Sun and Prof. Yuan designed the trial and were responsible for the data quality assurance, data analysis, manuscript writing, and manuscript publication. The steering committee oversaw the conduct, and participants safety of the trial. An independent data monitoring committee supervise the data quality and statistics. Outcome adjudication committee were blinded to treatment assignment, adjudicated all clinical events. The first three authors wrote the initial draft of the manuscript. All the authors edited the drafts of the manuscript and approved the submission of the manuscript. The principal investigators vouch for the accuracy and completeness of the data and for the fidelity of the trial to the protocol. The trial investigators and committees are listed in Supplementary data online, Appendix  *S1*. The study protocol has been published elsewhere.^22^ The trial was registered on Chictr.org (ChiCTR2100042238).

### Participants

We enrolled patients who met the inclusion criteria: (i) over 18 years of age; (ii) at least undergoing mitral valve or aortic valve surgery; (iii) without baseline AF and atrial flutter; (iv) with CHA_2_DS_2_-VASc score ≥2; (v) agreed to participate in the clinical study and provided written consent for telephone follow-ups.

We excluded patients who met any of the following exclusion criteria: (i) undergoing heart transplantation, complex congenital heart surgery or ventricular assist device implantation; (ii) redo cardiovascular surgery; (iii) left atrial diameter >6 cm; (iv) presence of thrombus in the left atrium or LAA; (v) with a history of stroke/cerebrovascular accident within 1 month before surgery.

Written informed consent was obtained from all the participants before enrolment.

### Procedures

The patients were randomly assigned in a 1:1 ratio, using a central randomization system, to undergo or not undergo SLAAO at the time of mitral or aortic valve surgery. A unique web-based central randomization system was developed specifically tailored for the three hospitals participating in the trial. This system ensured that randomization was conducted independently for each hospital, thereby eliminating any potential selection bias. The randomization plan was established by research staff not directly involved in the study. The surgeon team was not informed of the grouping until anaesthesia was administered.

In the SLAAO group, suture excision of the LAA was performed during the operation in addition to the index surgery. The LAA was amputated and its opening was sutured in two layers of polypropylene suture from the outside of the heart (see Supplementary data online, Appendix Supplementary data online, *Video S1*). LAA stump over one cm by intraoperative transoesophageal echocardiography (TEE) was defined as SLAAO failure. Intraoperative TEE was routinely performed to ensure the success of the occlusion. Additional manoeuvres were performed, during the index procedure, to rectify SLAAO failure. In the control group, the operation was performed according to the surgery plan without any LAA intervention. All surgical procedures were carried out by senior cardiac surgeons with volume more than 100 annual cases. All participants were required to adhere to the post-operative anticoagulation protocol after valve surgery (details see the protocol of Supplementary data online, Appendix).

Patients discharged alive were followed up by telephone or face-to-face interview at the time point of 1 month, 3 months, and 1 year. If the patients reported adverse events, the patient's medical records in the outpatient clinic were double checked. If the patients visit other hospitals, patients were required to send paper copies of medical records by mail or photocopies through the Internet.

### Outcomes

The primary outcome was a composite of newly occurred ischaemic stroke, transient ischaemic attack (TIA), and cardiovascular mortality during 1-year follow-up.

Secondary outcomes included POAF, cardiovascular mortality, newly occurred ischaemic stroke, newly occurred TIA, newly occurred haemorrhagic stroke, bleeding events of Bleeding Academic Research Consortium (BARC) type III, IV and V, and AF-associated healthcare utilization.

All the definitions of outcomes are provided in Supplementary data online, Appendix  *S2*).

### Statistical analyses

The primary hypothesis of the OPINION trial was that SLAAO added to usual care would reduce the risk of the composite primary outcome (ischaemic stroke, TIA, or cardiovascular mortality within 1 year) compared to usual care alone. Based on prior registries and clinical trials, the estimated event rate for the primary outcome in the control group (no SLAAO) was 6.8 per 100 person-years, accounting for variations in anticoagulant therapy and CHA₂DS₂-VASc scores. Assuming a 40% relative risk reduction in the SLAAO group, with 80% power at a one-sided significance level of 0.025 (equivalent to a two-sided 95% confidence interval [CI]), a total of 2118 participants (1059 per group) were required. This calculation incorporated potential loss-to-follow-up and heterogeneity in valvular surgery types (mechanical vs bioprosthetic/repair) and POAF incidence.

Data were collected and analysed according to the predefined statistical analysis plan. The main analysis was performed in accordance with the principles of intention-to-treat (ITT), which included all the randomized patients regardless of any occurrence of protocol deviation. Descriptive analysis was reported as frequencies (percentage [%]) for categorical variables and mean (standard deviation [SD]) or median (interquartile ranges [IQR]) for continuous variables. Categorical variables were compared between the two groups using the likelihood ratio χ² test or Fisher’s exact test. Continuous variables with normal distributions were compared using two sample *t*-tests, and non-normal continuous data were compared with the Wilcoxon rank-sum test. The 95% CIs of the difference between two treatment arms were calculated by normal approximation for continuous variables and by the Wald asymptotic method for binary variables. The event count and percentage of prespecified primary outcome and secondary outcomes were provided. The person-year rate for primary outcome was calculated and corresponding 95% CI was calculated by normal approximation method based on Poisson distribution. The time-to-first event rates for each group were estimated using Kaplan–Meier methods and were compared by the log-rank test. Proportional hazards assumptions of prespecified primary and secondary outcomes were examined by Schoenfeld residual test. Once proportional hazards assumption holds, between-group differences would be estimated by hazard ratios (HRs) with 95% CIs using a Cox proportional hazards model. If a patient experienced the same type of clinical event more than once, only the first one was used in the analysis. Superiority would be concluded if upper limit of the two-sided 95% CI of the HR in primary endpoint between groups was less than one. Sensitivity analysis of the primary outcome was done by multivariable Cox proportional hazards model with participating centres as a fixed effect. A landmark analysis was performed using 6 months as the landmark time point. Patients who remained event-free at 6 months was included in the subsequent analysis. The treatment effect would then be evaluated using a Cox proportional hazards model from the landmark time onward, and the HR with 95% CI were reported. Prespecified subgroup analyses were done with Cox proportional hazards regression models, presented as HRs with 95% CIs with *P*-values, and incorporated an interaction term. All analyses were performed with SAS software, version 9.4 (SAS Institute, Cary, NC). A two-sided *P*-value of <.05 was considered to indicate statistical significance.

## Results

### Participant characteristics

**Figure 1:**
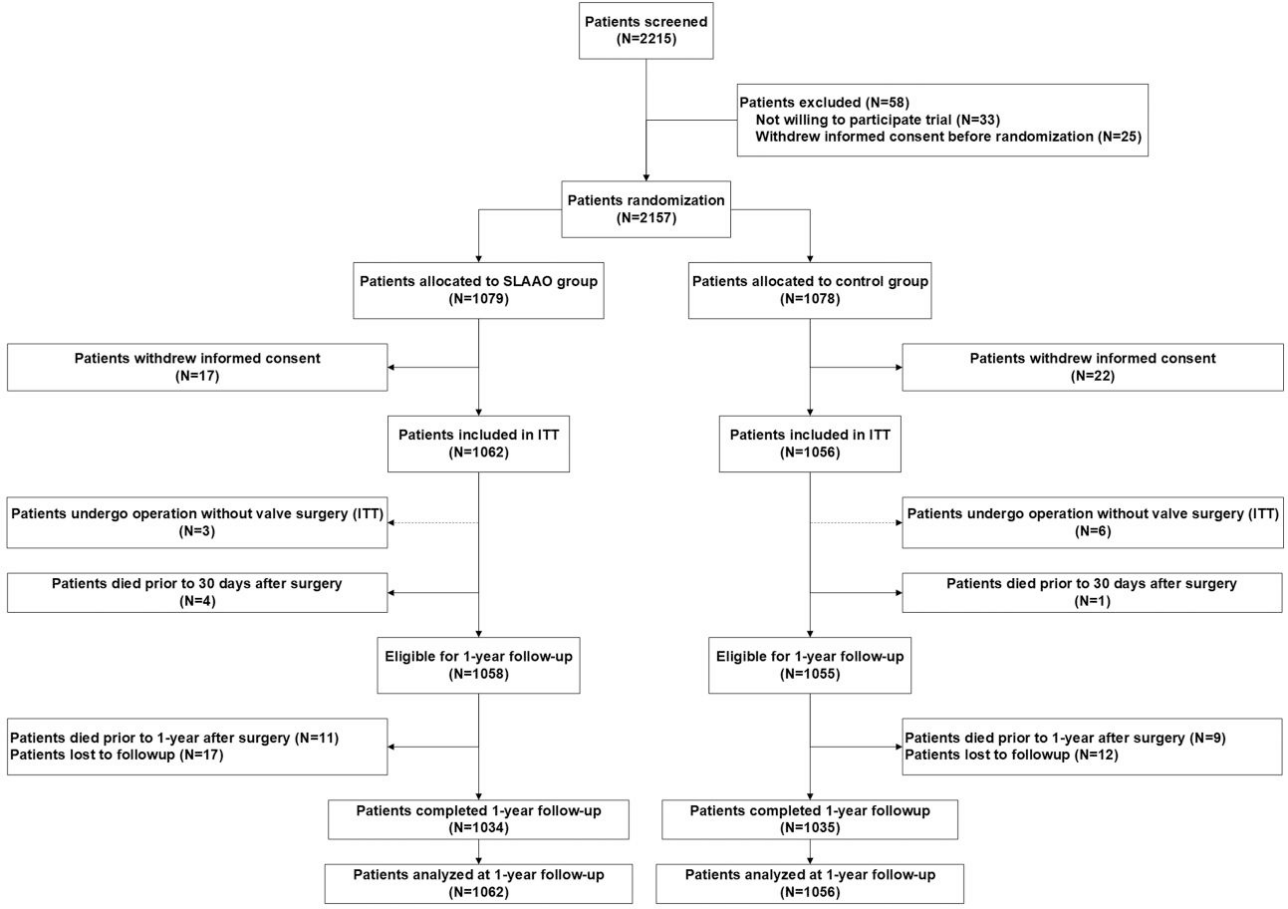
Flow diagram of the study. SLAAO, surgical left atrial appendage occlusion; ITT, intention-to-treat

Between April 2021 and June 2024, a total of 2157 participants from three centres were randomized to either undergo LAA occlusion (SLAAO: *n* = 1079) or no occlusion (control: *n* = 1078). After exclusion of 39 patients who withdrew informed consent, 2118 participants were included in the ITT population (1062 in the SLAAO group and 1056 in the control group). Final follow-up visits occurred from April 2022 to June 2025, with a mean follow-up duration of 1 year. Follow-up was completed by 98.6% of participants, and 1.4% (*n* = 29) were lost to follow-up (*Figure 1*).

Baseline characteristics were well-balanced between groups (*Table 1*). The mean age of participants was 55.6 (11.5) years, and 32.6% were female. The mean CHA_2_DS_2_-VASc score was 2.87 (0.97), and the mean EuroSCORE II was 1.57 (1.35)%. Intraoperative metrics were comparable: mean cross-clamp time was 92.4 min (SLAAO) vs 91.1 min (control) and mean cardiopulmonary bypass time was 124.0 vs 122.1 min. Reoperation for bleeding occurred in 1.1% (*n* = 12) of the SLAAO group and 1.7% (*n* = 18) of controls. Thirty-day mortality was 0.4% (*n* = 4) in the SLAAO group and 0.1% (*n* = 1) in controls (*Table 2*). Our analysis revealed no differences in anticoagulation and antiplatelet regimens between the two groups (see Supplementary data online, *Table S4.1*).

**Table 1:** Demographic and clinical characteristics of the participants at baseline and surgical treatments

Variables	SLAAO (*n* = 1062)	Control (*n* = 1056)	*P*-value
Participants			
Age (years), mean (SD)	55.5 (11.4)	55.6 (11.5)	.65
Female sex	349 (32.9%)	341 (32.3%)	.78
Smoking, former, or current	383 (36.1%)	383 (36.3%)	.92
Hypertension	492 (46.3%)	466 (44.1%)	.31
Hyperlipidaemia	170 (16.0%)	169 (16.0%)	1.00
Diabetes mellitus	139 (13.1%)	142 (13.4%)	.81
Previous stroke	130 (12.2%)	117 (11.1%)	.40
Transient ischaemic attack	218 (20.5%)	228 (21.6%)	.55
Previous myocardial infarction	102 (9.6%)	99 (9.4%)	.86
Peripheral arterial disease	146 (13.7%)	128 (12.1%)	.26
Aortic plaque	315 (29.7%)	317 (30.0%)	.86
History of heart failure	677 (63.7%)	683 (64.7%)	.66
Vascular disease	445 (41.9%)	433 (41.0%)	.67
Rheumatic heart disease	110 (10.4%)	110 (10.4%)	.96
CHA_2_DS_2_-VASc score, mean (SD)^a^	2.88 (0.98)	2.87 (0.96)	.83
EuroSCORE II (%), mean (SD)	1.58 (1.42)	1.56 (1.28)	.74
Cardiac surgery			
Valvular surgery type			.17
No valvular surgery	3 (0.3%)	6 (0.6%)	
Valve repair	322 (30.3%)	294 (27.8%)	
Mechanical valve replacement	576 (54.2%)	564 (53.4%)	
Bioprosthetic valve replacement	161 (15.2%)	192 (18.2%)	
Valvular disease			.29
Neither	3 (0.3%)	6 (0.6%)	
Aortic	527 (49.6%)	560 (53.0%)	
Mitral	414 (39.0%)	382 (36.2%)	
Aortic + Mitral	118 (11.1%)	108 (10.2%)	
Concomitant coronary artery bypass	271 (25.5%)	272 (25.8%)	.90
Any aortic procedure	165 (15.5%)	172 (16.3%)	.64

Data are numbers (%) unless stated otherwise. Baseline characteristics are presented for ITT population (*n* = 2118).

^a^Scores on the CHA_2_DS_2_-VASc scale reflect the risk of stroke and thrombo-embolism; scores range from 0 to 9, with higher scores indicating greater risk.

SLAAO, surgical left atrial appendage occlusion; SD, standard deviation; EuroSCORE, European System for Cardiac Operative Risk Evaluation; ITT, intention-to-treat.

**Table 2:** Trial outcomes

Outcome	SLAAO (*n* = 1062)	Control (*n* = 1056)	Comparison	*P*-value
Primary				
Newly occurred ischaemic stroke/TIA and cardiovascular mortality	73 (6.9%)	87 (8.2%)	0.83 (0.61, 1.14)	.25
Secondary				
Cardiovascular mortality	9 (0.9%)	8 (0.8%)	1.13 (0.43, 2.92)	.81
Newly occurred ischaemic stroke	26 (2.5%)	26 (2.5%)	1.00 (0.58, 1.72)	.99
Newly occurred TIA	40 (3.9%)	54 (5.2%)	0.74 (0.49, 1.11)	.14
Newly occurred haemorrhagic stroke	0 (0.0%)	3 (0.3%)	NA (NA, NA)	NA
Bleeding events of BARC type IV^b^	12 (1.1%)	18 (1.7%)	−0.58 (−1.68, 0.53)	.26
Bleeding events of BARC type III and V	1 (0.1%)	3 (0.3%)	0.33 (0.04, 3.20)	.34
Post-operative atrial fibrillation^b^	310 (29.2%)	341 (32.3%)	−3.10 (−7.12, 0.92)	.12
AF-associated health utilization	91 (8.8%)	100 (9.6%)	0.91 (0.68, 1.20)	.50
Operative				
Bypass time—min^ab^	124.0 (52.8)	122.1 (51.1)	1.90 (−2.53, 6.34)	.40
Cross-clamp time—min^ab^	92.41 (41.8)	91.08 (40.9)	1.33 (−2.20, 4.86)	.46
Post-operative				
Ventilation time—hour^ab^	17.2 (37.8)	16.9 (32.7)	0.24 (−2.77, 3.25)	.88
Length of post-operative hospital stay (day)^ab^	7.72 (3.7)	7.87 (3.5)	−0.15 (−0.46, 0.16)	.33
Post-operative acute kidney injury^b^	29 (2.7%)	25 (2.4%)	0.36 (−1.07, 1.80)	.60
Post-operative newly occurred ischaemic stroke^b^	13 (1.2%)	9 (0.9%)	0.37 (−0.59, 1.33)	.40
Reoperation for bleeding^b^	12 (1.1%)	18 (1.7%)	−0.58 (−1.68, 0.53)	.26
Perioperative IABP implantation^b^	4 (0.4%)	2 (0.2%)	0.19 (−0.36, 0.74)	.69
Perioperative ECMO implantation^b^	2 (0.2%)	0 (0.0%)	0.19 (−0.17, 0.54)	.50
Death within 30 days^b^	4 (0.4%)	1 (0.1%)	0.28 (−0.23, 0.79)	.37

Data are numbers (%) unless stated otherwise. All values are hazard ratios with 95% confidence intervals unless otherwise noted.

^a^Data are means (SD).

^b^This value is the difference with 95% confidence interval.

SLAAO, surgical left atrial appendage occlusion; TIA, transient ischaemic attack; NA, not available; AF, atrial fibrillation; IABP, intra-aortic balloon pump; ECMO, extra-corporeal membrane oxygenation; SD, standard deviation.

### Primary outcome

**Figure 2:**
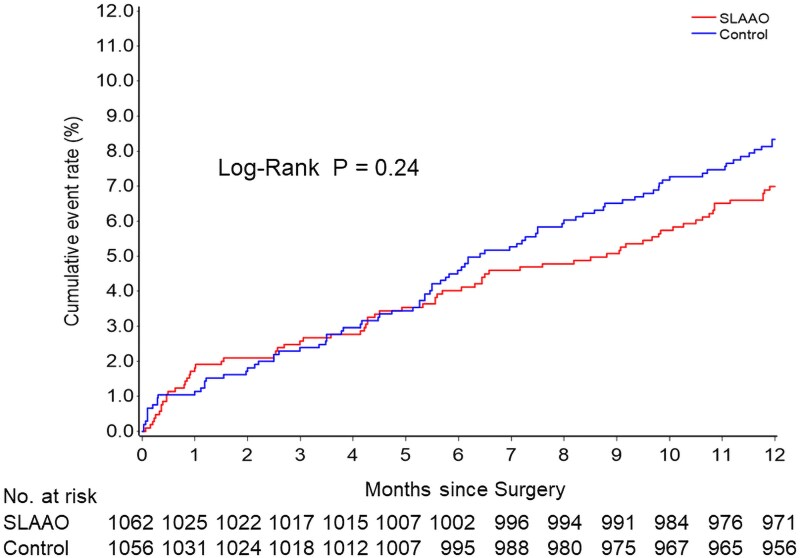
Cumulative incidence of newly occurred ischaemic stroke or transient ischaemic attack or cardiovascular mortality. SLAAO, surgical left atrial appendage occlusion

The primary composite endpoint occurred in 6.9% (*n* = 73) of the SLAAO group and 8.2% (*n* = 87) of controls (HR 0.83, 95% CI 0.61–1.14; *P* = .25) (*Table 2*). Kaplan–Meier analysis showed no significant difference in event-free survival between groups (log-rank *P* = .24; *Figure 2*). Landmark analyses at 6 months and 6–12 months showed results consistent with the overall findings (see Supplementary data online, *Table S4.2*).

### Secondary outcomes

During the 1-year follow-up, the incidence of newly occurred ischaemic stroke was identical in both groups (2.5% [*n* = 26] in SLAAO vs 2.5% [*n* = 26] in controls; HR 1.00, 95% CI 0.58–1.72), while TIAs were numerically lower in the SLAAO group (3.9% [*n* = 40] vs 5.2% [*n* = 54]; HR 0.74, 95% CI 0.49–1.11). Cardiovascular mortality rates were similar (0.9% [*n* = 9] vs 0.8% [*n* = 8]; HR 1.13, 95% CI 0.43–2.92).

POAF occurred in 29.2% (*n* = 310) of SLAAO patients compared to 32.3% (*n* = 341) of controls (*P* = .12), and AF-related healthcare utilization was marginally reduced with SLAAO (8.8% [*n* = 91] vs 9.6% [*n* = 100]; HR 0.91, 95% CI 0.68–1.20). No significant differences were observed in haemorrhagic stroke (0% vs 0.3%, *P* = .12), bleeding events of BARC type IV (1.1% vs 1.7%, *P* = .26), or bleeding events of BARC types III and V (0.1% vs 0.3%, *P* = .34) (*Table 2*).

### Subgroup and sensitivity analyses

**Figure 3:**
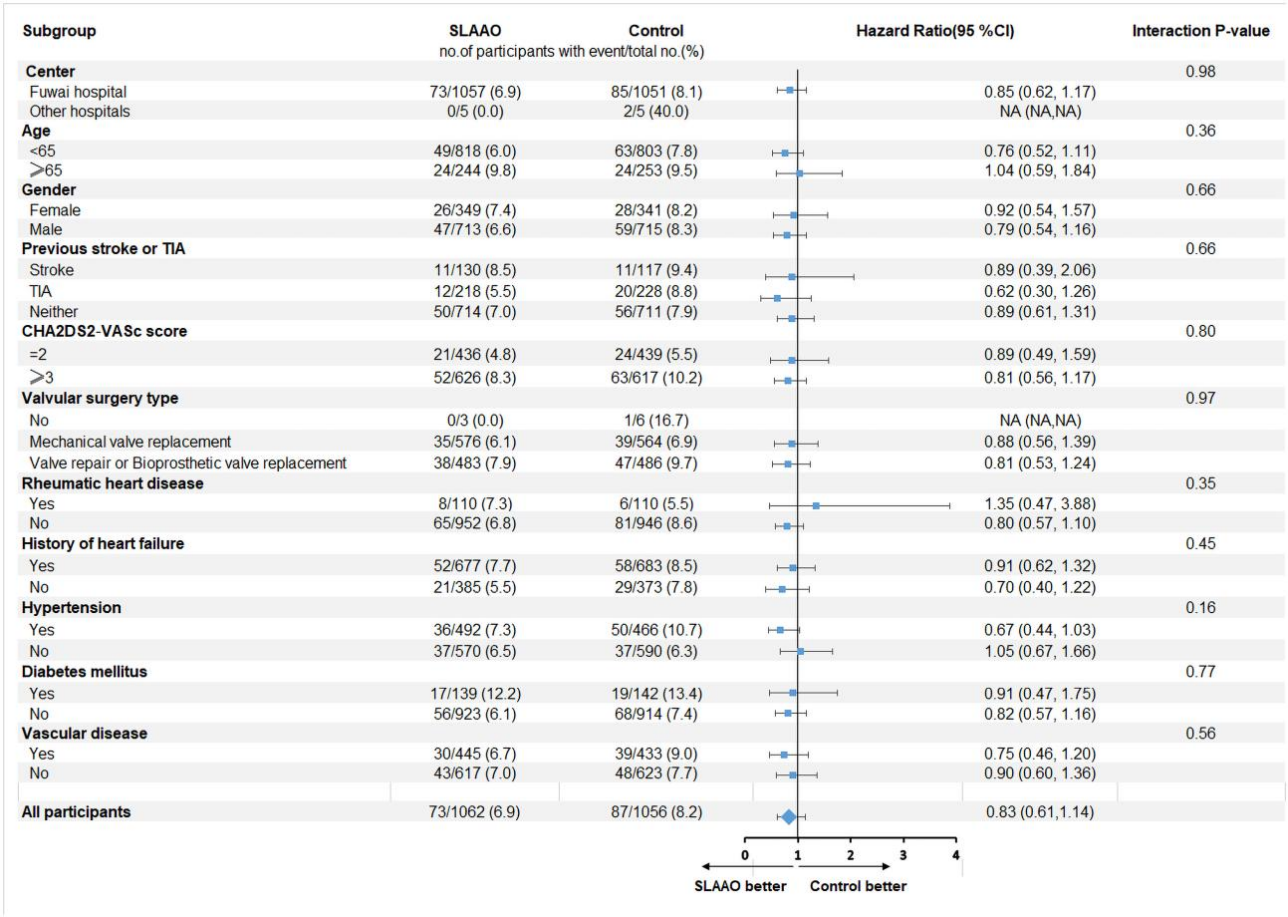
Forest of primary outcome during 1 year. Subgroup analysis of the effect of surgical left atrial appendage occlusion on primary outcome. SLAAO, surgical left atrial appendage occlusion; CI, confidence interval; TIA, transient ischaemic attack; Scores on the CHA_2_DS_2_-VASc scale reflect the risk of stroke and thrombo-embolism; scores range from 0 to 9, with higher scores indicating greater risk

Subgroup analyses revealed no significant interaction effects (all *P* for interaction >0.05; Supplementary data online, *Table S4.3* and *Figure 3*). The primary outcome rate is 7.3 per 100 person-years in the SLAAO group and 8.7 per 100 person-years in the control group (see Supplementary data online, *Table S4.4*). Sensitivity analyses of primary outcomes adjusting for centre effects yielded consistent results (HR 0.83, 95% CI 0.61–1.14) (see Supplementary data online, *Table S4.5*). Proportional hazards assumption test of outcomes also revealed no significance (all *P* > .05; Supplementary data online, *Table S4.6*).

### Safety outcomes

No significant differences were observed in death within 30 days (0.4% vs 0.1%, *P* = .37), haemorrhagic stroke (0% vs 0.3%, *P* = .12), bleeding events of BARC type IV (1.1% vs 1.7%, *P* = .26), or bleeding events of BARC type III and V (0.1% vs 0.3%, *P* = .34) (see Supplementary data online, *Table S3*).

## Discussion

This randomized controlled trial evaluated the clinical value of prophylactic SLAAO during valvular surgery in patients with CHA₂DS₂-VASc scores ≥2 but no pre-operative AF. Our primary findings demonstrated no significant difference in the incidence of the composite primary endpoint (newly occurred ischaemic stroke/TIA and cardiovascular mortality) between the SLAAO group and control group at 1-year follow-up (6.9% vs 8.2%, HR 0.83 [95% CI: 0.61–1.14], *P* = .25) (*Structured Graphical Abstract*). This key finding carries important clinical implications, suggesting that routine SLAAO may not reduce thrombo-embolic risk in this specific population at 1 year and that clinical decision-making requires more nuanced, individualized assessment.

When compared with prior research, our results demonstrate both consistencies and divergences. The landmark study using administrative data and involving 75 782 patients reported no significant thrombo-embolic reduction with LAA closure in non-AF subgroups.^21^ Another study demonstrated, in 1050 patients with sinus rhythm undergoing mitral valve repair for leaflet prolapse, that SLAAO did not significantly reduce the 5-year risk of TIA/stroke compared to leaving the LAA open, suggesting the need for further large-scale studies to clarify its role.^20^ However, Gercek *et al*.'s propensity score-matched study (*n* = 243) suggested a 67% stroke risk reduction in high CHA₂DS₂-VASc patients.^17^ These discrepancies likely arise from key methodological differences such as study design, surgical population, and unbalanced antithrombotic management protocols.

The relationship between SLAAO and POAF risk remains controversial in current literature.^17,19,23–25^ In a recent meta-analysis of six eligible studies, three investigations (total *n* = 1290) specifically evaluating POAF risk were pooled for analysis.^19^ These studies encompassed various occlusion techniques including direct suture, surgical amputation, and clip application. The combined results demonstrated no significant increase in POAF risk (pooled risk ratio 1.05, 95% CI 0.86–1.28; *P* = .63), with moderate heterogeneity observed (*I*² = 44.2%).^19^ Our findings corroborate this evidence, showing comparable POAF incidence between intervention and control arms.

Notably, despite the elevated incidence of POAF in high CHA₂DS₂-VASc score patients (30.7% in our cohort), consistent with the established range of 15%–54% reported in literature,^13–16,26^ SLAAO failed to demonstrate the anticipated protective effect against thromboembolism in the current study. This result, coupled with the comparable POAF rates between SLAAO and control groups (29.2% vs 32.3%, *P* = .12), suggests that POAF-related mechanisms may not be the primary driver of thromboembolism in this specific population.

This finding may be explained by several pathophysiological mechanisms:

First, cardiac surgery itself induces endothelial injury and inflammation,^27^ while valve prostheses create flow turbulence,^28^ both promoting thrombosis independent of AF presence/absence. Second, emerging evidence suggested that early post-operative AF primarily reflects systemic inflammatory responses,^29^ where platelet activation and hypercoagulability play more prominent roles than atrial mechanical dysfunction in thromboembolism pathogenesis. Third, the LAA's endocrine role is critical, as it serves as a major reservoir of atrial natriuretic peptide (ANP).^30^ Its exclusion during procedures (e.g. Maze procedure) has been linked to significant ANP reduction,^31^ which may disrupt sodium-water homeostasis and vascular tone regulation, potentially contributing to a prothrombotic state.^32,33^

The methodological strengths of this study include exceptional follow-up rates (98.6% completion), comprehensive quality control measures featuring systematic pre-operative, and post-operative transoesophageal echocardiographic evaluation with protocol-mandated surgical revision for residual LAA stumps >1 cm, and procedure standardization through exclusive involvement of high-volume surgeons (>100 annual cases).

However, several limitations should be acknowledged. First, the 1-year follow-up period represents a limitation. The observed divergence of event curves beyond 6 months suggests possible late effects that would require longer assessment, particularly in non-AF patients. Our ongoing prespecified 3- and 5-year follow-up evaluations will better characterize the intervention's clinical effects over time. Second, the study's power calculation assumed a 40% risk reduction based on registry data, but the observed 17% effect suggests SLAAO's benefit may be more modest in broader valvular surgery populations than previously estimated. The higher control-group event rate (8.2% vs projected 6.8%) further indicates our original assumptions may not fully account for real-world thromboembolic risks in these patients. Third, over half of the enrolled patients underwent mechanical valve replacement, necessitating lifelong warfarin therapy, which may have attenuated the potential benefits of SLAAO by independently reducing thromboembolic risk. Although sensitivity analysis did not reveal a statistically significant interaction between valve type (bioprosthetic vs mechanical) and SLAAO efficacy, the widespread use of anticoagulation in this subgroup could have obscured a true treatment effect. This underscores the need for further investigation in populations with limited or time-bound anticoagulation regimens, such as those receiving bioprosthetic valves or valve repairs. Fourth, uneven patient enrolment distribution (99.5% from a single centre), which was mitigated by uniform inclusion/exclusion criteria, standardized operative protocols, and centralized endpoint adjudication. Fifth, although anticoagulation status was not included as a prespecified subgroup in this trial, we plan to conduct *post hoc* analyses examining potential interactions between SLAAO and different antithrombotic regimens, which may generate hypotheses for future research. Finally, the potential impacts of SLAAO on cardiac function and neuroendocrine regulation remain uncharacterized and warrant future investigation.

## Conclusion

For valvular surgery patients with CHA₂DS₂-VASc scores ≥2 but no pre-operative AF, prophylactic SLAAO did not significantly reduce the incidence of the primary composite endpoint (ischaemic stroke, TIA, and cardiovascular mortality) at 1-year follow-up.

## Supplementary Material

ehaf674_Supplementary_Data

## Declarations

### Data Availability

The research data and statistical code will be available on http://www.medresman.org.cn/login.aspx in 6 months after the publication of the research results. To access the materials, authorization is required by contacting the study leader at drsunhs@sina.com.

### Pre-registered Clinical Trial Number

Left atrial appendage occlusion for patients with valvular diseases and without atrial fibrillation: a randomized controlled trial (ChiCTR2100042238, 2021-01-17, https://www.chictr.org.cn/).
