# Supplementary material for: Surgical left atrial appendage occlusion in valvular heart disease without atrial fibrillation: the OPINION trial
Source: Eur Heart J. 2025 Sep 1;47(20):2440–8. doi: 10.1093/eurheartj/ehaf674 (PMC13191831; doi:10.1093/eurheartj/ehaf674)
Supplement: ehaf674_Supplementary_Data [file ehaf674_supplementary_data.zip › Supplement0806.doc]

SUPPLEMENTAL TEXT, TABLES, AND REFERENCES

**Surgical left atrial appendage occlusion in valvular heart disease without atrial fibrillation: the OPINION trial**

**TABLE OF CONTENTS**

S1. Details of the OPINION Collaborative Group	2

S2. Definition of Outcomes*.	4

S3. Adverse Events and Serious Adverse Events.	6

S4. Results of Sensitivity Analysis and Subgroup Analysis	7

References	12

## S1. Details of the OPINION Collaborative Group

**Writing Committee**

Xin Yuan (M.D., Ph.D., Professor, Fuwai Hospital, Beijing), Ju Fan (M.D., Ph.D., Fuwai Hospital, Beijing), Hengchao Wu (M.D., Ph.D., Associate Professor, Fuwai Hospital, Beijing), Yanyan Zhao (MSc., Fuwai Hospital, Beijing), Xin Wang (M.D., Ph.D., Professor, Fuwai Hospital, Beijing), Sheng Liu (M.D., Ph.D., Professor, Fuwai Hospital, Beijing), Xianqiang Wang (M.D., Ph.D., Professor, Fuwai Hospital, Beijing), Junming Zhu (M.D., Ph.D., Professor, Anzhen Hospital, Beijing), Pixiong Su (M.D., Ph.D., Professor, Chaoyang Hospital, Beijing), Fei Xu (M.D., Ph.D., Professor, Fuwai Hospital, Beijing), Wei Feng (M.D., Ph.D., Professor, Fuwai Hospital, Beijing), Yan Yang (M.D., Ph.D., Professor, Fuwai Hospital, Beijing), Yang Wang (Ph.D, Professor, Fuwai Hospital, Beijing), Hansong Sun (M.D., Ph.D., Professor, Fuwai Hospital, Beijing)

**Steering Committee**

Hansong Sun (Principal Investigator, Fuwai Hospital, Beijing), Xin Yuan (Fuwai Hospital, Beijing), Junming Zhu (Anzhen Hospital, Beijing), Pixiong Su (Chaoyang Hospital, Beijing), Yan Zhang (Fuwai Hospital, Beijing)

**Data Monitoring Committee**

Yang Wang (Chair, Fuwai Hospital, Beijing), Yanyan Zhao (Fuwai Hospital, Beijing)

**Outcome Adjudication Committee (Auditing Committee)**

Yan Zhang (Chair, Fuwai Hospital, Beijing), Jun Feng (Fuwai Hospital, Beijing), Tao Yang (Fuwai Hospital, Beijing),

**Central Coordinating Centre (Fuwai Hospital, Beijing)**

Administration: Xin Yuan, Fan Ju, Hengchao Wu, Baotong Li

Clinical: Hansong Sun, Xin Yuan, Hengchao Wu, Xin Wang, Sheng Liu, Xianqiang Wang, Fei Xu, Wei Feng, Yan Yang;

Data Management: Fan Ju, Zeyi Yin

IT Support: Mengnan Zhu;

Principal Investigator: Hansong Sun;

Statistics: Yanyan Zhao, Wang Yang.

**Local Clinical Centres**

Anzhen Hospital, Beijing (Junming Zhu, Chenhan Zhang)

Chaoyang Hospital, Beijing (Pixiong Su, Haibin Li)

## S2. Definition of Outcomes*.

| **Outcomes** | **Definitions** |
| --- | --- |
| Cardiovascular Mortality | Cardiovascular death is defined as any death, during 1-year follow up, with a cardiovascular cause and includes those deaths occurring within 30 days of a cardiovascular procedure (e.g. cardiac surgery, percutaneous transluminal coronary angioplasty), cardiac arrest, myocardial infarction, pulmonary embolus, stroke, hemorrhage, or deaths due to an unknown cause conformed by adjudication commitee.^1^ |
| Newly Occurred Ischemic Stroke | Acute focal neurological dysfunction caused by focal infarction at single or multiple sites of the brain. Evidence of acute infarction may come either from a) symptom duration lasting more than 24 hours, or b) neuroimaging or other technique in the clinically relevant area of the brain. The term does not include infarction of the retina. (ICD-11 8B11) |
| Newly Occurred TIA | Transient episode of focal neurological dysfunction caused by focal brain ischemia without acute infarction in the clinically relevant area of the brain or transient monocular visual loss due to retinal ischemia. Symptoms should resolve completely within 24 hours. (ICD-11 8B10) |
| POAF | New-onset atrial fibrillation (≥30 seconds on ECG or Holter) occurring during hospitalization or within 30 days post-surgery, in patients without prior history of atrial fibrillation.^2^ |
| Newly Occurred Hemorrhagic Stroke | Acute neurological dysfunction caused by haemorrhage within the brain parenchyma or in the ventricular system. (ICD-11 8B00) |
| Bleeding Events | BARC type III/IV/V bleeding events, such as any transfusion with overt bleeding, cardiac tamponade, bleeding requiring surgical intervention for control (excluding dental/nasal/skin/hemorrhoid), bleeding requiring intravenous vasoactive agents, intracranial hemorrhage (does not include microbleeds or hemorrhagic transformation, does include intraspinal), subcategories confirmed by autopsy or imaging or lumbar puncture intraocular bleed compromising vision, perioperative intracranial bleeding within 48 h, reoperation after closure of sternotomy for the purpose of controlling bleeding, transfusion of ≥5U whole blood or packed red blood cells within a 48-h period, chest tube output ≥2L within a 24-h period, or fatal bleeding.^3^ |
| Atrial Fibrillation Associated Health Utilization | It is measured by the event rates of outpatient visits and hospitalizations with a diagnosis of Atrial Fibrillation.^2^ |
| ICD: International Classification of Diseases; TIA: Transient Ischemic Attack; POAF: Postoperative Atrial Fibrillation; ECG: Electrocardiograph; BARC: Bleeding Academic Research Consortium. | |

*All events were adjudicated by an independent committee blinded to treatment allocation.

## S3. Adverse Events and Serious Adverse Events.

|  | **SLAAO**  **(N=1062)** | **Control**  **(N=1056)** | **P Value** |
| --- | --- | --- | --- |
|  | **No. events/no. total (%)** | |  |
| **Adverse Events** |  |  |  |
| Bleeding events of BARC type IV | 12 (1.1%) | 18 (1.7%) | 0.26 |
| **Serious Adverse Events** |  |  |  |
| Death within 30 postoperative days | 4 (0.4%) | 1 (0.1%) | 0.37 |
| Newly occurred hemorrhagic stroke | 0 (0.0%) | 3 (0.3%) | NA |
| Bleeding events of BARC type III, and V | 1 (0.1%) | 3 (0.3%) | 0.34 |

SLAAO: Surgical Left Atrial Appendage Occlusion; BARC: Bleeding Academic Research Consortium; NA: Not Available.

## S4. Results of Sensitivity Analysis and Subgroup Analysis

| **Table S4.1 Anticoagulation and antiplatelet therapy during follow-up** | | | | |
| --- | --- | --- | --- | --- |
|  | **Total (N=2069)*** | **SLAAO (N=1034)** | **Control (N=1035)** | **P value** |
| Following prespecified medication protocol# |  |  |  | 0.42 |
| Lifelong warfarin therapy | 874 (42.2%) | 448 (43.3%) | 426 (41.2%) |  |
| Warfarin therapy for 6 months | 660 (31.9%) | 320 (30.9%) | 340 (32.9%) |  |
| Aspirin + Lifelong warfarin therapy | 239 (11.6%) | 112 (10.8%) | 127 (12.3%) |  |
| Aspirin + Warfarin therapy for 6 months | 287 (13.9%) | 151 (14.6%) | 136 (13.1%) |  |
| Prespecified medication violation |  |  |  |  |
| Only aspirin therapy | 9 (0.4%) | 3 (0.3%) | 6 (0.6%) |  |
| Temporary interruption of protocol specified medications |  |  |  | 0.50 |
| Pause aspirin therapy due to bleeding | 1 (0.04%) | 1 (0.1%) | 0 (0.0%) |  |
| Short-term warfarin therapy for paroxysmal AF after 6 months | 12 (0.6%) | 5 (0.5%) | 7 (0.7%) |  |
| Short-term NOAC therapy for paroxysmal AF after 6 months | 4 (0.2%) | 1 (0.1%) | 3 (0.3%) |  |
| *: After the exclusion of 49 participants who died or were lost to follow-up, the medication information of 2069 participants during follow-up were collected.  #: Medication violation was defined as deviation from the prespecified protocol for >20% of total follow-up time. In the trial, 9 participants met this criterion for medication violation.  SLAAO: Surgical Left Atrial Appendage Occlusion; NOAC: Non-Vitamin K Antagonist Oral Anticoagulants | | | | |

| **Table S4.2 Landmark analysis result of primary outcome：Overall and valve surgery type subgroup** | | | | | | | | | |
| --- | --- | --- | --- | --- | --- | --- | --- | --- | --- |
|  | **0-6 month** | | | |  | **6-12 month** | | | |
|  | **SLAAO** | Control | HR  (95% CI) | **P value** |  | **SLAAO** | **Control** | **HR**  **(95% CI)** | **P value** |
| Overall | 42/1062  (4.0%) | 48/1056  (4.5%) | 0.87  (0.58,1.32) | 0.52 |  | 31/1002  (3.1%) | 39/995  (3.9%) | 0.79 (0.49,1.26) | 0.31 |
| Cox proportional hazards model was used to calculate hazard ratio and corresponding 2-sided 95% confidence interval between SLAAO and Control. Kaplan-Meier survival analysis was used to calculate event rate in each group.  SLAAO: Surgical Left Atrial Appendage Occlusion; HR: Hazard Ratio; CI: Confidence Interval. | | | | | | | | | |

| **Table S4.3 Subgroup analysis results of primary outcomes#** | | | | |
| --- | --- | --- | --- | --- |
|  | **SLAAO**  **(N=1062)** | **Control**  **(N=1056)** | **Hazard Ratio and 95% CI** | **P Value** |
| Age |  |  |  |  |
| <65 year | 49/818 (6.0%) | 63/803 (7.8%) | 0.76 (0.52,1.11) | 0.15 |
| >=65 year | 24/244 (9.8%) | 24/253 (9.5%) | 1.05 (0.59,1.84) | 0.88 |
| Gender |  |  |  |  |
| Female | 26/349 (7.4%) | 28/341 (8.2%) | 0.92 (0.54,1.57) | 0.75 |
| Male | 47/713 (6.6%) | 59/715 (8.3%) | 0.79 (0.54,1.16) | 0.24 |
| Previous stroke or TIA |  |  |  |  |
| Stroke | 11/130 (8.5%) | 11/117 (9.4%) | 0.89 (0.39,2.06) | 0.79 |
| TIA | 12/218 (5.5%) | 20/228 (8.8%) | 0.62 (0.30,1.26) | 0.18 |
| Neither | 50/714 (7.0%) | 56/711 (7.9%) | 0.89 (0.61,1.31) | 0.56 |
| CHA2DS2‑VASc score* |  |  |  |  |
| =2 | 21/436 (4.8%) | 24/439 (5.5%) | 0.89 (0.49,1.59) | 0.68 |
| >=3 | 52/626 (8.3%) | 63/617 (10.2%) | 0.81 (0.56,1.17) | 0.26 |
| Valve surgery type |  |  |  |  |
| No valve surgery | 0/3 (0.0%) | 1/6 (16.7%) | NA (NA, NA) | NA |
| Mechanical valve replacement | 35/576 (6.1%) | 39/564 (6.9%) | 0.88 (0.56,1.39) | 0.58 |
| Valve repair or Bioprosthetic valve replacement | 38/483 (7.9%) | 47/486 (9.7%) | 0.81 (0.53,1.25) | 0.34 |
| Rheumatic heart disease |  |  |  |  |
| Yes | 8/110 (7.3%) | 6/110 (5.5%) | 1.35 (0.47,3.88) | 0.58 |
| No | 65/952 (6.8%) | 81/946 (8.6%) | 0.80 (0.57,1.10) | 0.17 |
| History of heart failure |  |  |  |  |
| Yes | 52/677 (7.7%) | 58/683 (8.5%) | 0.91 (0.62,1.32) | 0.61 |
| No | 21/385 (5.5%) | 29/373 (7.8%) | 0.70 (0.40,1.22) | 0.21 |
| Hypertension |  |  |  |  |
| Yes | 36/492 (7.3%) | 50/466 (10.7%) | 0.67 (0.44,1.03) | 0.06 |
| No | 37/570 (6.5%) | 37/590 (6.3%) | 1.05 (0.67,1.66) | 0.83 |
| Diabetes mellitus |  |  |  |  |
| Yes | 17/139 (12.2%) | 19/142 (13.4%) | 0.91 (0.47,1.75) | 0.77 |
| No | 56/923 (6.1%) | 68/914 (7.4%) | 0.82 (0.57,1.16) | 0.26 |
| Vascular disease |  |  |  |  |
| Yes | 30/445 (6.7%) | 39/433 (9.0%) | 0.75 (0.47,1.20) | 0.23 |
| No | 43/617 (7.0%) | 48/623 (7.7%) | 0.90 (0.60,1.36) | 0.63 |
| #:Cox proportional hazards model was used to calculate hazard ratio and corresponding 2-sided 95% confidence interval between SLAAO and Control. Kaplan-Meier survival analysis was used to calculate event rate in each group  *: Scores on the CHA2DS2‑VASc scale reflect the risk of stroke among patients with atrial fibrillation; scores range from 0 to 9, with higher scores indicating greater risk.  SLAAO: Surgical Left Atrial Appendage Occlusion; CI: Confidence Interval; TIA: Transient Ischemic Attack; NA: Not Available. | | | | |

| **Table S4.4 Person-year rates of primary outcome during 1 year** | | | | | | |
| --- | --- | --- | --- | --- | --- | --- |
| **Indicator** | **SLAAO**  **(N=1062)** | | **Control**  **(N=1056)** | | **Rate difference and 95%CI^#^** | **P value*** |
|  | **Event No.** | **Rate/ per 100 person-years^#^** | **Event No.** | **Rate/ per 100 person-years^#^** |  |  |
| Primary outcome during 1 year | 73 | 7.3  （5.6, 8.9） | 87 | 8.7  （6.9, 10.6） | -1.5  （-3.9, 1.0） | 0.25 |
| #: 95% confidence interval was calculated by normal approximation method based on Poisson distribution.  *: P value for superiority test.  SLAAO: Surgical Left Atrial Appendage Occlusion; CI: Confidence Interval. | | | | | | |

| **Table S4.5 Sensitivity analysis of primary outcome** | | | |
| --- | --- | --- | --- |
|  | **SLAAO (N=1062）** | **Control（N=1056）** | **Hazard Ratio/ Rate Difference and 95%CI** |
|  | **No. of participants (%)** | |  |
| Newly occurred ischemic stroke/transient ischemic attack and cardiovascular mortality |  |  |  |
| Cox proportional hazards with centre adjustment^#^ | 73 (6.9%) | 87 (8.2%) | 0.83 (0.61, 1.14) |
| Kaplan-Meier Method* | 73 (6.9%) | 87 (8.2%) | -1.35% (-3.63%, 0.93%) |
| #: Cox proportional hazards model with center adjustment was used to calculate hazard ratio and corresponding 2-sided 95% confidence interval between SLAAO and Control.  *: Kaplan-Meier survival analysis was used to calculate event rate differences and corresponding 2-sided 95% confidence interval between SLAAO and Control.  SLAAO: Surgical Left Atrial Appendage Occlusion; CI: Confidence Interval. | | | |

| Table S4.6 Proportional Hazards assumption test of outcomes | |
| --- | --- |
| Outcomes | Scheonfeld residuals test p value |
| Primary Outcomes | 0.66 |
| Secondary Outcomes |  |
| Cardiovascular mortality | 0.39 |
| Newly occurred ischemic stroke | 0.63 |
| Newly occurred transient ischemic attack | 0.49 |
| Newly occurred hemorrhagic stroke | 1.00 |
| Bleeding events | 0.28 |
| AF-associated health utilization | 0.73 |
| AF: atrial fibrillation |  |

## References
